# *Streptococcus gordonii* DL1 evades polymorphonuclear leukocyte-mediated killing via resistance to lysozyme

**DOI:** 10.1371/journal.pone.0261568

**Published:** 2021-12-20

**Authors:** Yumiko Urano-Tashiro, Keitarou Saiki, Yuki Yamanaka, Yuiko Ishikawa, Yukihiro Takahashi

**Affiliations:** Department of Microbiology, Nippon Dental University School of Life Dentistry at Tokyo, Tokyo, Japan; Universidade Estadual de Ponta Grossa, BRAZIL

## Abstract

*Streptococcus gordonii* is an etiological bacterial agent of infective endocarditis. Although the pathogenesis mechanisms are not well understood, the interaction between streptococci and phagocytes is considered important for the development of infective endocarditis. Previous studies show that some *S*. *gordonii* strains, including DL1, survive in polymorphonuclear leukocytes (PMNs), whereas other strains such as SK12 are sensitive to PMN-dependent killing. In this study, we assessed the differences between the sensitivity of *S*. *gordonii* DL1 and *S*. *gordonii* SK12 to PMN-dependent killing. *S*. *gordonii* DL1 showed a higher survival when treated with PMNs than SK12. Both *S*. *gordonii* DL1 and *S*. *gordonii* SK12 showed high resistance to low pH condition. Compared to *S*. *gordonii* SK12, *S*. *gordonii* DL1 was sensitive to hydrogen peroxide. However, the resistance of *S*. *gordonii* DL1 to the tested bactericidal agents, especially lysozyme, was higher than that of SK12. Furthermore, we performed a bactericidal assay by treating a mixture of *S*. *gordonii* DL1 and SK12 with PMNs. *S*. *gordonii* DL1 did not enhance the survival of *S*. *gordonii* SK12 exposed to PMNs. These results indicated that *S*. *gordonii* DL1 is resistant to bactericidal agents that degrade bacteria in phagolysosomes. In addition, there was no secretory factor involved in the resistance to bactericidal agents. The findings of this study may help develop treatments for infective endocarditis caused by *S*. *gordonii*.

## Introduction

Oral streptococci, including *Streptococcus gordonii*, are components of the normal microbial flora of the human oral cavity [1]. In addition, these streptococci colonize damaged heart valves and are recognized as etiological bacterial agent of infective endocarditis (IE) [2–4]. The pathogenesis of IE depends on various distinct virulence determinants. Previous investigations have focused on the contributions of specific adhesive interactions [5]. We have previously reported that the sialic acid-binding adhesin (Hsa) of *S*. *gordonii* DL1 contributes to the pathogenesis of IE [6]. In addition, the Hsa adhesin and its homologues facilitate attachment of *S*. *gordonii* to host cells such as polymorphonuclear leukocytes (PMNs), erythrocytes, platelets, macrophages, and monocytes [7–16].

Considerable differences have been observed in the virulence of representative *S*. *gordonii* strains in the rat models of IE [17]. Lee *et al*. reported that *S*. *gordonii* DL1 induces the development of severe endocarditis, whereas *S*. *gordonii* SK12 does not cause any disease [17]. Furthermore, pathogenic strains, including DL1, are resistant to PMN-dependent killing. In contrast, non-pathogenic strains, including SK12, are highly sensitive to PMN-dependent killing.

IE development depends not only on the colonization and proliferation of bacteria in the endocardium, but also on the ability of bacteria that enter the blood stream to travel to damaged heart valves while escaping immune cells. Thus, the ability of *S*. *gordonii* to resist or avoid host cellular and humoral defenses may represent an important virulence determinant in IE pathogenesis. There is a difference in the susceptibility among *S*. *gordonii* strains to the bactericidal activity of human PMNs [17]. However, the mechanism by which streptococci survive in the phagosomes of PMNs is not well understood. The aim of this study was to determine the potential mechanisms associated with the differences in resistance between *S*. *gordonii* DL1 and SK12 to PMN-mediated killing, and determine the potential mechanisms associated with resistance.

## Materials and methods

### Ethics statement

All experiments involving human participants have been approved by our Institutional Review Board. Healthy donors were informed of the study protocol and they provided written consent for using the collected samples. Collection and use of blood samples in this study was approved by the Research Ethics Committee of Nippon Dental University (NDU-T2016-10).

### Bacterial strains and growth conditions

The *S*. *gordonii* strains used in this study were DL1 (Challis strain) and SK12 [10, 18]. Streptococci were cultured overnight at 37°C in brain heart infusion (BHI) broth (BD Biosciences, Franklin Lakes, NJ, USA).

### Isolation of human PMNs

Human PMNs were isolated from peripheral blood collected form healthy donors as previously described [9]. Erythrocytes were removed via dextran segmentation and hypotonic lysis. PMNs were washed three times and resuspended in Roswell Park Memorial Institute (RPMI)-1640 medium (Nissui Pharmaceutical, Tokyo, Japan) containing 1% bovine serum albumin, 0.2% HEPES, and 0.15 mM CaCl_2_ (supplemented RPMI medium).

### Bactericidal assay

Killing of bacteria by human PMNs was measured via a colony formation assay [19]. Streptococci were pre-incubated with 2% anti-Hsa antibody [9] for 30 min at room temperature, thereafter that added 20% autologous serum (from the same donor from whom PMNs were isolated) was added and incubated for 30 min at room temperature, it was then washed, and resuspended in supplemented RPMI medium. Briefly, PMNs (5×10^6^ cells) and the opsonized bacteria (1×10^6^ cells) were suspended in 1 ml supplemented RPMI medium, mixed, and incubated in 1.5-mL tubes for 1 and 2 h at 37°C with mixing using a rotator. The PMNs were disrupted with sterile water at room temperature, diluted with RPMI medium, and plated on BHI agar. Colonies were counted after incubation of the plates at 37°C for 2 days. Percent survival was calculated based on number of colony forming units of PMNs-mixed bacteria compared to that of bacteria alone. For certain experiments, we used *S*. *gordonii* DL1 harboring plasmids that confer spectinomycin resistance and SK12 harboring plasmids that confer erythromycin resistance [20]. These DL1 and SK12 strains were cultured on BHI plates containing 200 μg/ml spectinomycin (Sigma-Aldrich, St Louis, MO, USA) or 10 μg/ml erythromycin (Sigma-Aldrich).

### LIVE/DEAD experiment

Intracellular bacterial viability was observed *in situ* by examining exclusion of a fluorescent dye using the LIVE/DEAD^TM^ BacLight^TM^ Bacterial Viability kit (Molecular Probes, Eugene, OR, USA) [19, 21]. Briefly, both PMNs (1×10^6^ cells) and the opsonized bacteria (1×10^7^ cells) were suspended in 1 ml supplemented RPMI medium, mixed, and incubated in 1.5-mL tubes for 2 h at 37°C with mixing using a rotator. A fluorescent dye mixture was added to the PMN-opsonized bacterial mixture to yield to a final concentration of 5 μM SYTO 9 and 30 μM propidium iodide, and the mixture was incubated for 15 min at room temperature. Subsequently, 3 μl of the mixture was placed on a glass slide, covered with a coverslip, and examined using a fluorescence microscope (LSM800; Carl Zeiss, Oberkochen, Germany). Bacteria with intact cell membranes appeared green due to staining with SYTO 9, whereas bacteria with damaged cell membranes were stained red with propidium iodide.

### pH tolerance assay

Overnight cultures of *S*. *gordonii* were harvested and resuspended in RPMI medium (pH 7.0) and then 50 μl (5×10^5^ cells) of bacteria were transferred to 1ml of RPMI medium (pH 3.0, 4.0, or 5.0). After 30 and 120 min of incubation at 37°C, samples were serially diluted and plated on BHI agar. Percent survival was calculated as described above.

### Hydrogen peroxide (H_2_O_2_) tolerance assay

Overnight cultures of *S*. *gordonii* were harvested and resuspended in RPMI medium (pH 7.0), and then 5×10^5^ cells/ml of bacteria were transferred to RPMI medium (pH 5.0) supplemented with H_2_O_2_ (2.5, 5.0, or 10 mM). After 30 and 120 min of incubation at 37°C, samples were serially diluted and plated on BHI agar. Percent survival was calculated as described above.

### Bactericidal agents resistance assay

Overnight cultures of *S*. *gordonii* were harvested and resuspended in RPMI medium (pH 7.0), and then 5×10^5^ cells/ml of bacteria were transferred to RPMI medium (pH 7.0 or 5.0) supplemented with lysozyme (FUJIFILM, Osaka, Japan) (5 mg/ml), defensin (PEPTIDE, Osaka, Japan) (5 μg/ml), lactoferrin (Sigma-Aldrich) (10 μg/ml), or a mixture of these bactericidal agents. After 2 h of incubation at 37°C, samples were serially diluted and plated on BHI agar. Percent survival was calculated as described above.

### Statistical analysis

Statistically significant differences of the means of obtained values were evaluated by unpaired *t*-test using *P* < 0.05 as the threshold for significance.

## Results

### Survival of *S*. *gordonii* DL1 compared to that of *S*. *gordonii* SK12 when treated with human PMNs

**Fig 1:**
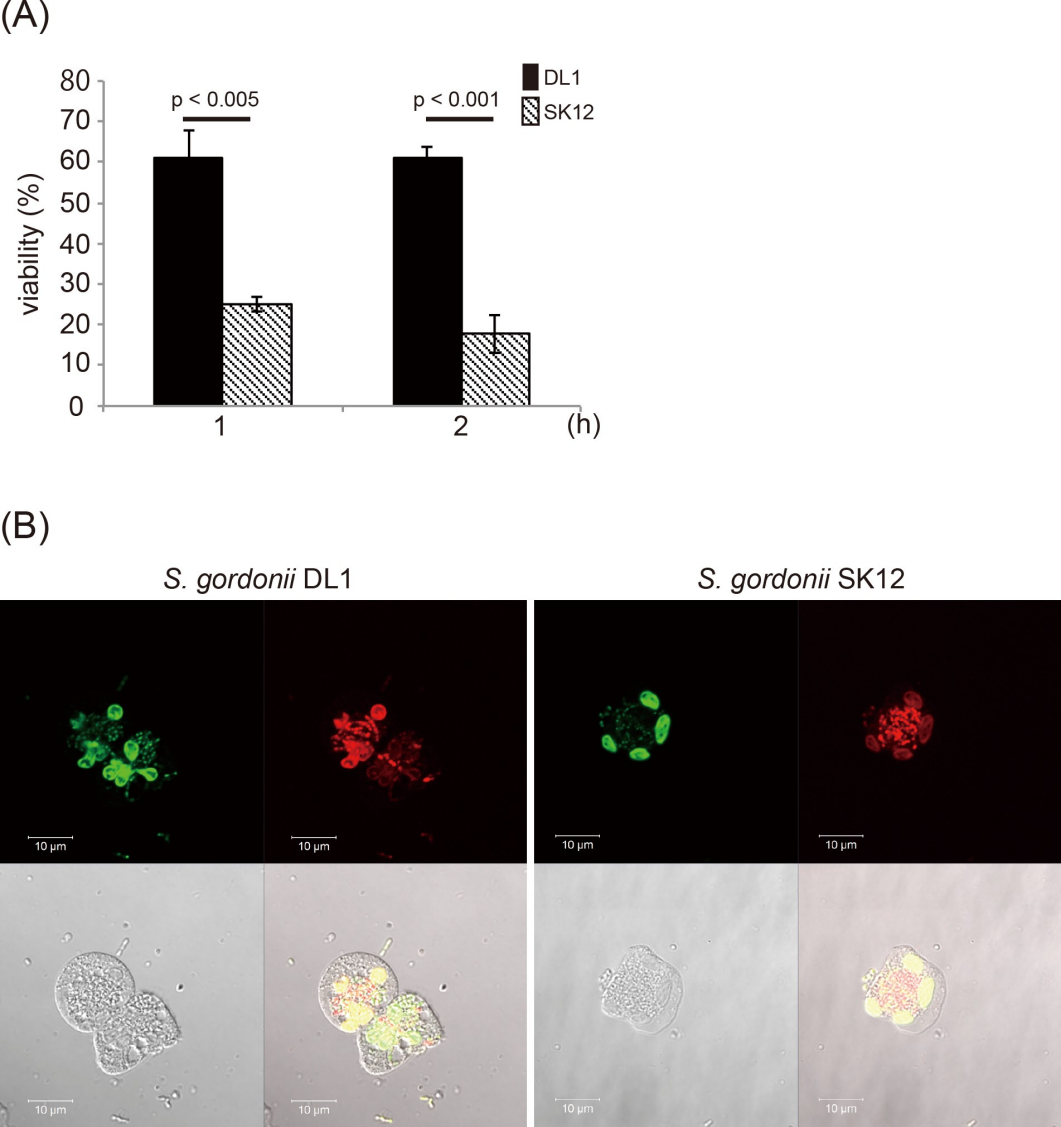
Bactericidal assay of *Streptococcus gordonii* strains treated with human polymorphonuclear leukocytes (PMNs). (A) *Streptococcus gordonii* DL1 (1×10^6^ cells) and SK12 (1×10^6^ cells) were incubated with or without human PMNs (5×10^6^ cells) for 1 or 2 h at 37°C on a rotator. The PMN/bacteria mixture was disrupted with sterile water and plated on BHI agar plates to enumerate live bacteria. Percent survival was calculated based on the number of colony forming units of bacteria mixed with PMNs compared to that of bacteria alone. The standard error is marked with error bars (n = 4). Statistical differences of the means of obtained values were evaluated by unpaired *t*-test. (B) *S*. *gordonii* DL1 and SK12 were exposed to PMNs for 2 h and stained with SYTO 9 and propidium iodide. Representative fluorescence micrographs of *S*. *gordonii* DL1 (a) and SK12 (b) are shown. Scale bar = 10 μm. Green indicates live cells. Red indicates dead cells.

A previous study reported differences between the susceptibility of various *S*. *gordonii* strains to human PMNs [17]. Therefore, we first investigated the survival of *S*. *gordonii* DL1 and SK12 treated with PMNs. Human PMNs were incubated for 2 h with *S*. *gordonii* DL1 or SK12 at a 1:5 ratio of bacterial cells: host cells. The susceptibility of *S*. *gordonii* strains was assessed via a colony formation assay. As shown in Fig 1A, a considerable PMN-dependent killing of bacteria was observed in reaction mixtures containing *S*. *gordonii* SK12 (82.8% killing of added *S*. *gordonii* SK12 at 2 h). Under identical conditions, the proportion of dead of *S*. *gordonii* DL1 was significantly lower (39%) than that of SK12. Moreover, we evaluated the integrity of the bacterial membrane inside PMNs *in situ* via staining with fluorescent dyes (Fig 1B). Representative fluorescence micrographs of *S*. *gordonii* DL1 showed that most of the intracellular bacteria were stained green with STYO 9, indicating that most of the bacterial cells were alive (Fig 1B). In contrast, the majority of intracellular *S*. *gordonii* SK12 were stained red with propidium iodide (Fig 1B). These data suggest that the sensitivity of *S*. *gordonii* SK12 to PMN-dependent killing was higher than that of *S*. *gordonii* DL1.

### Survival of *S*. *gordonii* strains under different conditions

**Fig 2:**
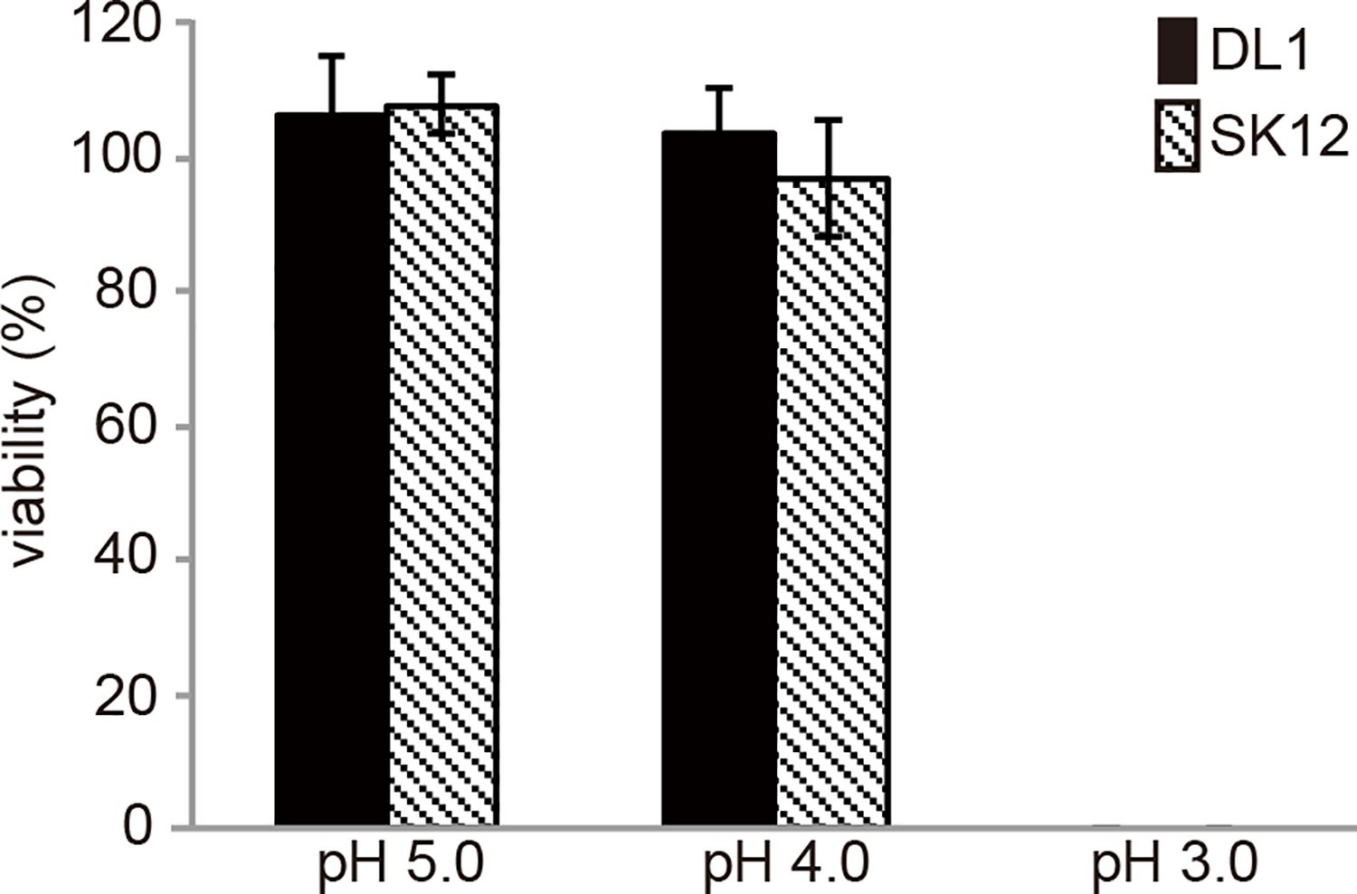
Survival of *Streptococcus gordonii* under acidic conditions. *Streptococcus gordonii* DL1 and SK12 were cultured in RPMI media of varied pH conditions for 2 h at 37°C. Percent survival was calculated based on number of colony forming units of bacteria in acidic conditions compared to that of bacteria cultured in medium at pH 7.0. n = 4; mean with standard error is shown. Statistical differences in the means of obtained values were evaluated via unpaired *t*-test.

*S*. *gordonii* DL1 and SK12 differ in resistance to PMN-meditated killing. Phagolysosomes contain different bactericidal factors, such as reactive oxygen species, acids, or enzymes to degrade bacterial cells [22]. We therefore examined the strain-dependent differences under various bactericidal conditions. First, we evaluated the ability of the strains to survive under low pH conditions (pH 5.0, 4.0, and 3.0); however, we found no differences in survival at low pH after 2 h (Fig 2). Both *S*. *gordonii* DL1 and SK12 could survive in acidic medium at pH 4.0. No living bacterial cells were detected in the medium at pH 3.0. Since the phagosomal pH is 5.0 [22], we used an acidic medium (pH 5.0) for subsequent H_2_O_2_ and enzymatic lysis resistance assays.

**Fig 3:**
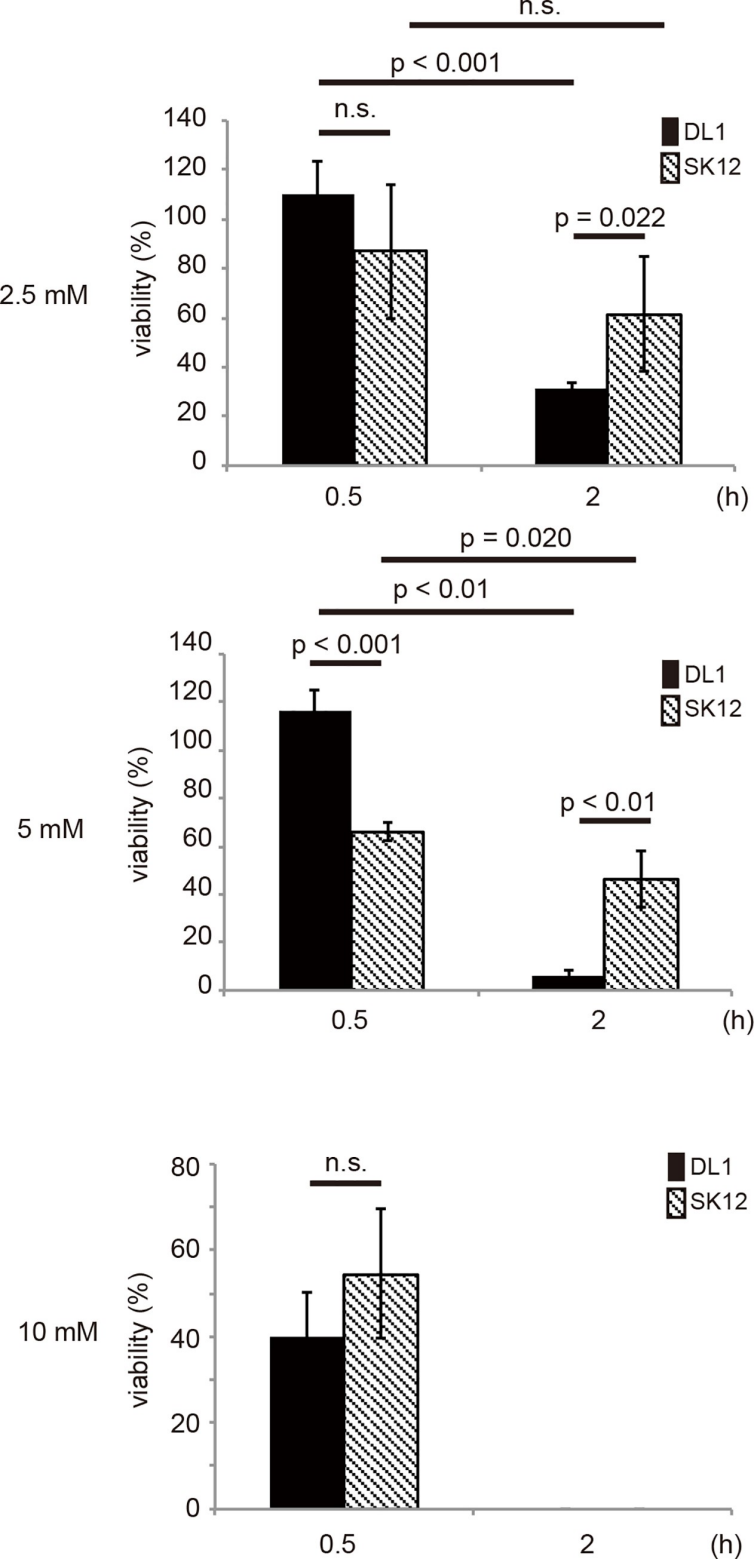
Survival of *Streptococcus gordonii* in the presence of H_2_O_2_. *Streptococcus gordonii* DL1 and SK12 were cultivated in medium at pH 5.0 supplemented with hydrogen peroxide (H_2_O_2_) at different concentrations for 30 min or 2 h at 37°C. Percent survival was calculated based on the number of viable cells in H_2_O_2_-supplemented medium compared to that in medium without H_2_O_2_. n = 4; mean with standard error is shown. Statistical differences in the means of obtained values were evaluated via unpaired *t*-test. n.s., not significant.

To test the resistance to H_2_O_2_-mediated degradation, *S*. *gordonii* DL1 and SK12 were treated with different concentrations of H_2_O_2_ in RPMI medium at pH 5.0 for 30 min or 2 h (Fig 3). *S*. *gordonii* DL1 survived in medium supplemented with 2.5 mM and 5 mM H_2_O_2_ after 30 min of incubation. However, the growth of *S*. *gordonii* DL1 was highly impaired after 2 h of incubation; only 31.5% (2.5 mM H_2_O_2_) and 6.1% (5 mM H_2_O_2_) viable bacterial cells were found compared to the number of control cells. The growth of *S*. *gordonii* SK12 decreased in medium containing H_2_O_2_ after 30 min incubation, 87.0% (2.5 mM) and 66.2% (5 mM) viable bacteria cells were found compared to the number of control cells. However, unlike in *S*. *gordonii* DL1, *S*. *gordonii* SK12 survived in medium containing H_2_O_2_ after 2 h of incubation. It was found that 61.5% (2.5 mM) and 46.2% (5 mM) of *S*. *gordonii* SK12 cells survived compared to the number of control cells. The use of 10 mM H_2_O_2_ for 30 min resulted in a survival of 39.8% for *S*. *gordonii* DL1 and 54.6% for *S*. *gordonii* SK12 compared to the number of control cells. No live bacterial cells were detected in medium supplemented with 10 mM H_2_O_2_ at 2 h. These results suggest that the sensitivity of *S*. *gordonii* DL1 to H_2_O_2_ was higher than that of *S*. *gordonii* SK12.

### Survival of *S*. *gordonii* strains in the presence of bactericidal agents

**Fig 4:**
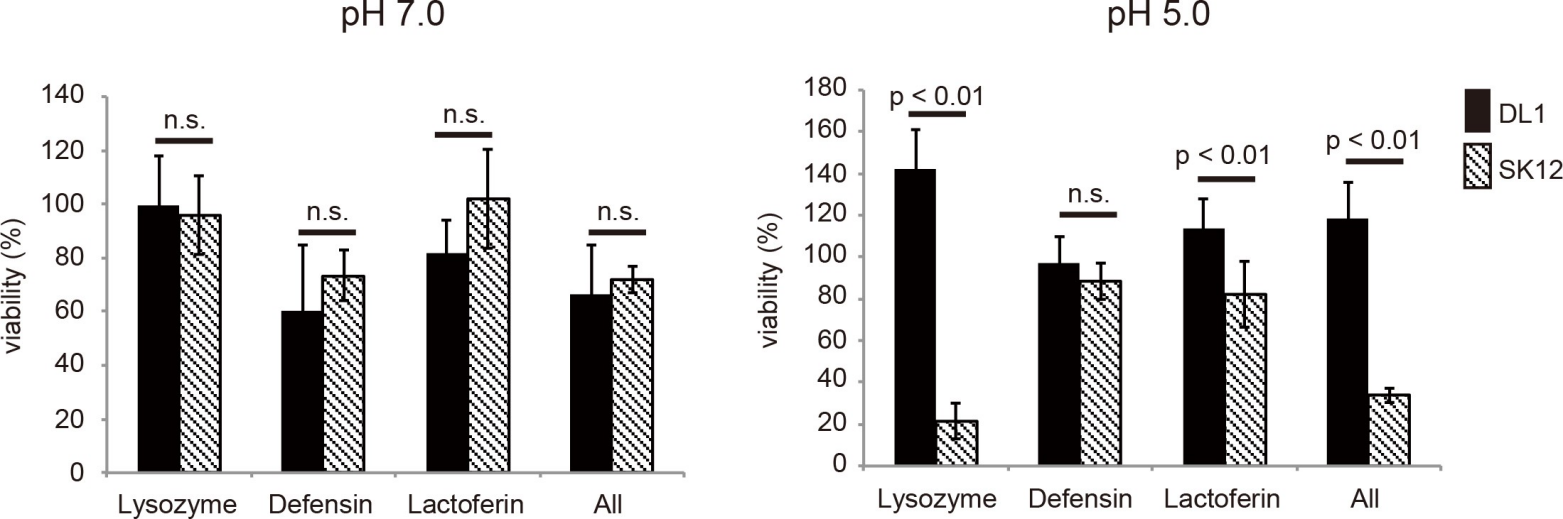
Survival of *Streptococcus gordonii* in bactericidal agent supplemented RPMI medium. *Streptococcus gordonii* DL1 and SK12 were cultivated in medium containing lysozyme (5 mg/ml), defensin (5 μg/ml), Lactoferrin (10 μg/ml), or a mixture of the three reagents for 2 h at 37°C. Percent survival was calculated based on the number of viable cells in bactericidal agent-supplemented medium compared to that in medium without bactericidal agents. n = 4; mean with standard error is shown. Statistical differences in the means of obtained values were evaluated via unpaired *t*-test. n.s., not significant.

Next, we determined the sensitivity of *S*. *gordonii* strains to several bactericidal agents. When several bactericidal agents were treated for bacteria with medium at pH 7.0, we did not find any difference in survival (Fig 4). When we used the medium at pH 5.0, *S*. *gordonii* DL1 survived in the presence of lysozyme; 142% live cells were found compared to the number of control cells (Fig 4). In contrast, the growth of *S*. *gordonii* SK12 was inhibited by lysozyme. The viability of *S*. *gordonii* SK12 was 21.7% in the presence of lysozyme compared to the number of control cells. There were no significant differences between the growth of *S*. *gordonii* DL1 and SK12 in the presence of defensin; the viability of *S*. *gordonii* DL1 and SK12 was 97.2% and 88.4%, respectively, compared to the number of control cells. Lactoferrin treatment slightly inhibited the growth of *S*. *gordonii* SK12 to 82.1%, compared to the number of control cells. These data indicate that the susceptibility of *S*. *gordonii* SK12 to bactericidal agents, particularly lysozyme, was higher than that of *S*. *gordonii* DL1.

### Secretion factors are not required for resistance to anti-bactericidal activity

**Fig 5:**
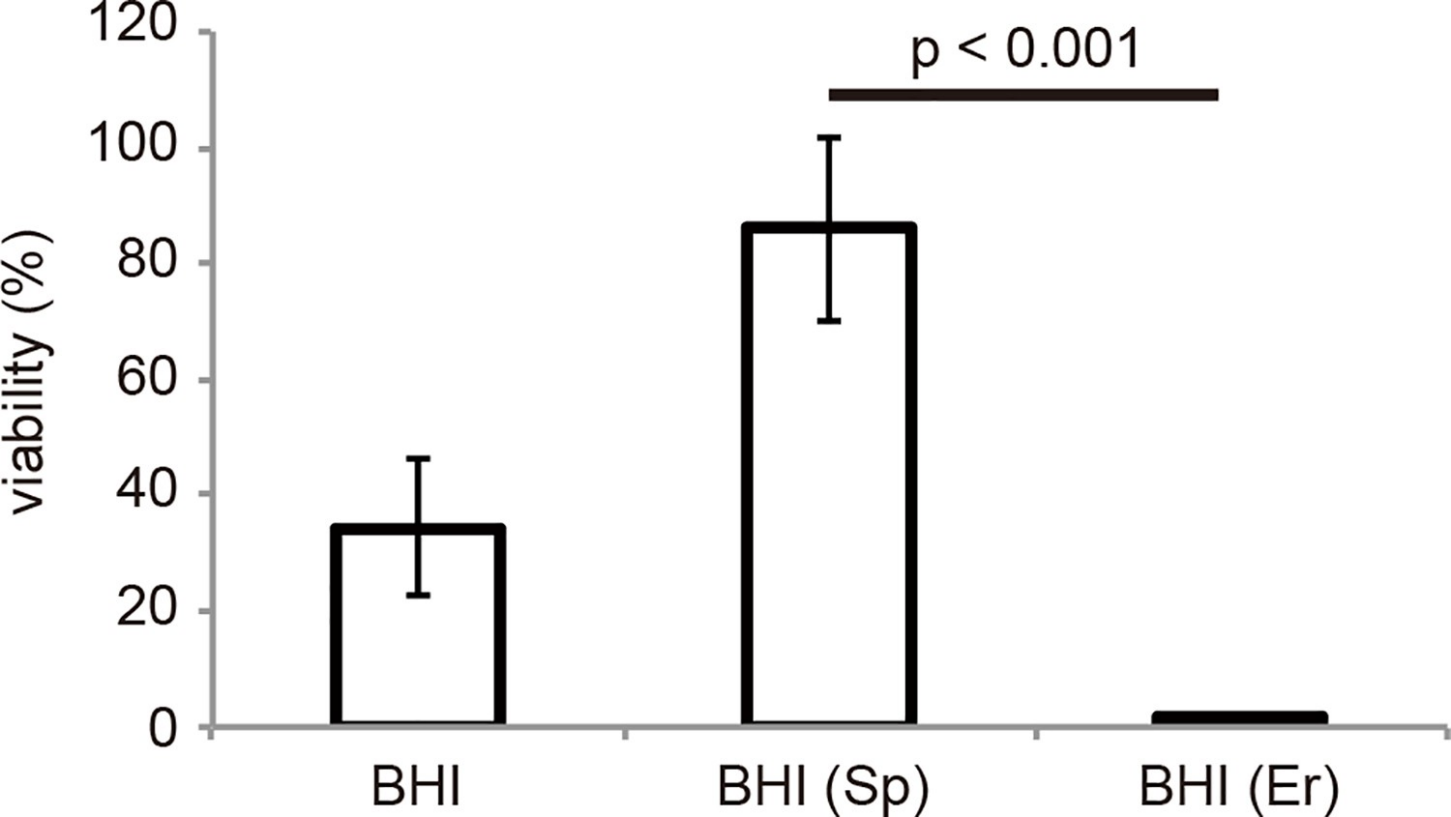
Survival of *Streptococcus gordonii* in human PMNs. For bactericidal assays, we prepared *Streptococcus gordonii* DL1 harboring a plasmid that conferred spectinomycin (Sp) resistance and *S*. *gordonii* SK12 harboring a plasmid that conferred erythromycin (Er) resistance. Human PMNs (1×10^6^ cells) were incubated with bacteria (4×10^6^ cells; 1:1 mixture of *S*. *gordonii* DL1 and SK12) for 2 h at 37°C on a rotator. The PMN/bacteria mixture was disrupted with sterile water and plated on BHI agar plates supplemented with or without Sp or Er to enumerate live bacteria. Percent survival was calculated based on the number of colony forming units of bacteria mixed with PMNs compared to that of bacteria alone. n = 4; mean with standard error is shown. Statistical differences in the means of obtained values were evaluated via unpaired *t*-test.

*S*. *gordonii* DL1 may produce some secretion factors to protect it from bactericidal agents. We expected that the factors secreted by *S*. *gordonii* DL1 could assist the survival of *S*. *gordonii* SK12 within PMNs. To confirm this, we performed bactericidal assays using antibacterial spectinomycin-resistant *S*. *gordonii* DL1 and erythromycin-resistant *S*. *gordonii* SK12 to discriminate both strains on drug containing BHI plates. Human PMNs were incubated for 2 h with *S*. *gordonii* DL1 and SK12 mixture at a 4:1 ratio of bacterial cells: PMNs (Fig 5). When PMNs were challenged with a mixture of both strains, approximately 36% of the bacterial cells survived compared to the number of control cells. However, *S*. *gordonii* DL1 accounted for most of the surviving cells (about 86%). There was no protective effect on *S*. *gordonii* SK12. This result indicates that no secretory factor was involved in the resistance of *S*. *gordonii* DL1 to bactericidal agents.

## Discussion

*S*. *gordonii* and related species of the viridans group of streptococci are also well known for the contribution to IE [2–4]. The ability of *S*. *gordonii* to evade the host immune response may represent an important factor for IE pathogenesis. However, the mechanism by which streptococci escape from the host immune defenses during the course of IE progression is not understood.

In the present study, we analyzed the effects of bactericidal agents which are characteristic of phagolysosomes on the survival of *S*. *gordonii* DL1 and SK12. Lee *et al*. revealed that pathogenic *S*. *gordonii* strains are resistant to PMN-dependent killing, whereas a large number of non-pathogenic *S*. *gordonii* strains are killed by PMNs [17].

Phagolysosomes have different bactericidal mechanisms to kill and degrade microbial pathogens. We investigated three of bactericidal effects to evaluate *S*. *gordonii* resistance and determine whether resistance can affect bacterial survival in phagolysosomes. *S*. *gordonii* strains survive in acidic conditions, which may be an advantage for survival in phagolysosomes. *S*. *gordonii*, like other viridans group streptococci, produces H_2_O_2_ via an NADH oxidase, which reduces molecular oxygen to H_2_O_2_ [23]. *S*. *gordonii* DL1 avoids degradation based on a combination of resistance to reactive oxygen species (ROS) and the capability to damage lysosomes/phagosomes within macrophages [24]. Our data showed that *S*. *gordonii* strains were resistant to H_2_O_2_-mediated degradation. During the initial treatment with H_2_O_2_ for 30 min, *S*. *gordonii* DL1 exhibited a survival rate higher than that of *S*. *gordonii* SK12. This could be attributed to the ability of *S*. *gordonii* DL1 to remove more of the generated superoxide from the solution than that by *S*. *gordonii* SK12 [24], and to the presence of non-catalase ROS resistance mechanisms in *S*. *gordonii* DL1 [25, 26]. However, during a longer incubation, such as 2 h, *S*. *gordonii* SK12 had a higher survival rate in the presence of H_2_O_2_ than that of *S*. *gordonii* DL1. Therefore, the resistance to H_2_O_2_ could be associated with the biological activity of the strains rather than with their specific resistance mechanisms against anti-bactericidal agents.

Neutrophil internalizes microbes into phagosomes, which then fuse with lysosomes to form phagolysosomes. Phagolysosome are acidified by proton pumps and matured. Lysosomal antimicrobial proteins such as lysozyme, lactoferrin, lipocalin, and gelatinase are activated in the acidic condition of phagosomes [27]. In phagolysosomes, microbes are killed by a combination of non-oxidative and oxidative mechanisms [28]. Our data indicate that *S*. *gordonii* DL1 may be more resistant to phagosomal enzymes, especially lysozyme, than *S*. *gordonii* SK12. Some pathogenic bacteria have evolved mechanisms to evade lysozyme-mediated killing by modifying their peptidoglycan [29]. For example, *Streptococcus pyogenes*, a group A streptococcus, lacking the peptidoglycan *N*-acetylglucosamine deacetylase A (*pgdA*) is more sensitive to killing by lysozyme in vitro and is less virulent in vivo [30, 31]. In *Staphylococcus aureus*, the *N*-acetylmuramic acid acetylation by *O*-acetyltransferase A (*oatA*) enhances resistance to lysozyme in vitro and bacterial survival in vivo [32, 33]. In addition, mutation of the phosphoglucosamine mutase (*glmM*) in *S*. *gordonii* DL1 appears to increases roughness of the bacterial cell surface and sensitivity to lysozyme [19, 34]. The resistance to bactericidal agents, particularly lysozyme, shown by *S*. *gordonii* DL1 in our study indicates that composition and/or structure of the cell wall might differ between *S*. *gordonii* DL1 and SK12. This may provide an advantage to *S*. *gordonii* DL1 for facilitating survival within PMNs.

*S*. *gordonii* DL1 was found to be resistant to bactericidal agents which killing and degrade pathogens in phagolysosomes. *S*. *pyogenes* produces several factors that enable survival in neutrophils after phagocytosis [35]. Streptococcal M and M-like proteins can prevent degranulation as well as phagosomal fusion of azurophilic granules [36]. *S*. *pyogenes* secretes streptolysin, a pore-forming toxin, to lyse neutrophils and other host cells [37]. However, *S*. *gordonii* DL1 lacks genes that encode homologues of these factors. Hence, we predicted that *S*. *gordonii* DL1 may produce another secretory factor to protect bacteria from bactericidal agents. We performed bactericidal assay in which human PMNs were incubated with *S*. *gordonii* DL1 and SK12 mixture. We expected that if *S*. *gordonii* DL1 secreted any factors to protect bacteria from bactericidal agents, it would protect *S*. *gordonii* SK12 from killing and degradation in phagosomes. However, the result suggests that no secretory factor may be involved in the survival of *S*. *gordonii* DL1 in PMNs. Further studies should determine the detailed mechanisms via which *S*. *gordonii* evades phagosomal degradation. The identification of genes that confer resistance to PMN-dependent killing may provide an important insight into the pathogenesis of IE and may facilitate the development of new drugs for the prevention of IE.
